# Stroke survivors*’* perceptions of living with disability in urban Ghana- a qualitative study

**DOI:** 10.1186/s12889-024-21030-6

**Published:** 2024-12-18

**Authors:** Petter Persson, Anna Hughton, Sibylle Herzig van Wees

**Affiliations:** 1 Department of Women’s and Children’s Health, Uppsala University, Dag Hammarskjölds väg 14B, Uppsala, Sweden; 2 Department of Physiotherapy, School of Biomedical and Allied Health Sciences College of Health Sciences, , University of Ghana Part-Time Lecture And Accra Physiotherapy & Sports Injury Clinic, Clinic Director/Physiotherapist, Accra, Ghana; 3 Department of Global Public Health, Karolinska Institutet, Stockholm, Sweden

**Keywords:** Disability, Stroke, Everyday life, Wellbeing, Ghana

## Abstract

**Background:**

Stroke prevalence is one of the most pressing issues in many sub-Saharan African countries. Stroke survivors often face disabilities, mental health problems, and experience stigma. Research on post-stroke experiences and interventions is limited, particularly in low and middle-income countries, including Ghana. This study aims to contribute to this gap by investigating experiences of everyday life following a stroke in urban Ghana.

**Method:**

A qualitative in-depth study involving 14 interviews with stroke survivors who attended physiotherapy at Accra Physiotherapy and Sports Injury Clinic. Qualitative analysis was conducted to analyse the data.

**Results:**

Data analysis generated four themes; [1] Mobility limitations; [2] Psychosocial burden [3] Employment Limitations [4] Financial burden.

**Conclusion:**

Stroke survivors with disabilities perceive themselves as non-functional members of society, which limits their options for a normal life and undermines their well-being. A more in-depth understanding of these issues can contribute to strategies, interventions, and policies that address these problems.

**Supplementary Information:**

The online version contains supplementary material available at 10.1186/s12889-024-21030-6.

## Introduction

Over one billion people globally bear some form of disability [1]. Of these individuals, 80% live in low-income countries [1]. The term disability is broad and includes several different types of impairments, including physical, visual, and hearing disabilities, among others [1]. Disability has been increasing globally due to an aging population and increase in non-communicable diseases (NCDs) [1]. Non-communicable diseases are estimated to account for 23 of the 25 leading causes of years lived with disability globally [2]. In sub-Saharan Africa, NCDs have developed into a growing epidemic and place significant pressure on healthcare systems [3]. With the rise in stroke incidences, it has been of particular concern to countries in sub-Saharan Africa, including Ghana [4–6]. Estimates show that around 2.6% of the Ghanaian population over 50 years of age suffer from stroke [7].

The increasing burden of stroke prevalence poses a major challenge for the Ghanaian health and social care system. Although Ghana has introduced a national health insurance authority (NHIA) [8, 9], inequalities in health financing remain a challenge, particularly for chronic health conditions [10]. Moreover, the risk of developing mental ill-health and depressive disorders has been reported as increasing substantially after experiencing a stroke, both globally and in Ghana [11–13]. Furthermore, Obembe et al. writes that depression after experiencing a stroke leads to worse possibilities regarding the ability to reintegrate into the community [14].

Moreover, mobility of those living with a disability is particularly challenging in a rapidly urbanising city like Accra [15]. Limited mobility can pose challenges to physical rehabilitation and social integration. Given the growing burden of stroke disease and the growing number of people living with disabilities following a stroke, more research is needed to understand their perceptions and needs to support effective recuperation and integration into society. Consequently, this study aims to better understand the perceptions of stroke survivors on living in urban Accra. This study investigated four areas: mental health and well-being; perceptions of physical mobility their views on rehabilitation and reintegration and the financial burden of stroke.

## Materials and methods

### Aim

: The aim of this research was to explore and understand how stroke survivors with disabilities perceive life in urban Accra.

### Primary research question

What are the perceptions of stroke survivors with disabilities regarding their everyday life in urban Accra?

### Research Objectives

- Exploring the perceptions of stroke survivors of their mobility and spatial environment in Accra.
- Exploring the perceptions of stroke survivors of their social activities and community in Accra.
- Exploring the perceptions of stroke survivors of their perceptions of reintegration into society in Accra.

### Study design

This was a qualitative study using in-depth interviews to explore participants’ individual experiences [16]. This study was conducted between December 2019 and February 2020 and recruitment took place at a private physiotherapy clinic in Accra, Ghana. The study population consisted of patients coming either with a referral from a doctor or with a self-referral to obtain rehabilitation post-stroke.

### Sampling and recruitment

#### Sampling

Purposive sampling was applied in consideration of specific inclusion and exclusion criteria [17]. There was also an element of convenience sampling since the participants were all patients at a specific clinic at a certain time and therefore were convenient to interview [17]. The participants that met the inclusion criteria were asked to participate in the study. Three participants were excluded because they could not comprehend information orally in English. Furthermore, one individual chose not to participate in the study. In total, 14 interviews were completed.

### Inclusion criteria

For inclusion, the participant must be a stroke survivor and understands English. Participants in the study needed to have a physical disability related to stroke that they defined as a physical hindrance related to mobility in their daily life.

### Recruitment

The recruitment process was conducted at Accra Physiotherapy and Sport Injury Clinic, with assistance from several local physiotherapists. All patients who met the inclusion criteria and attended the clinic (14 in total) received a brief information letter about the study. Those interested in participating and meeting the inclusion criteria were provided with further details on the study’s procedures. Recruitment was led by the first author, supported by the second author and local physiotherapists. In total, 14 patients received the information letters, which the first author distributed along with verbal explanations about the study.

Both a welcome letter, and an information letter were distributed to participants, containing information relating to the study and how collected data was to be used. These documents also emphasized the participants right to leave the study at any time. Furthermore, a letter of consent was given out to the participants to sign. Participants were advised to look through all the information and give informed consent if they wanted to be part of the study. Consent for recording the interviews was accepted in written form on the study documents. There was also an opportunity to give consent through fingerprints which were used by one of the participants. Participants were given as much time as they needed to consider participating in the study. The interviews began with an explanation of the interview and its purpose and ended with a thank-you to the participants.

### Fieldwork procedure

Participants were approached with the support of local physiotherapists who initiated the contact and consents were sought. A semi-structured interview-guide was used to conduct the interviews. The interview guide was developed only for this study. Interviews took between 30 and 45 min. The interviews were conducted by the first author (PP) over ten weeks in 2019/2020. The interview-guide development was based on collaboration across the research team. The guide was adapted following feedback from local physiotherapists in Ghana to make sure the questions were culturally suitable. Two pilot interviews were performed to further improve the interview guide.

### Ethical consideration

The project received ethical approval from the Ghana Health Service Ethics Review Committee (GHS-ERC020/11/19). We followed the Helsinki Declarations, provided all study information in advance, obtained informed written consent. All data was anonymised and is stored according to local regulations. For ethical reasons, interviews were conducted at the clinic, participants’ homes, or offices to minimize any financial or time burden. Interview timing was determined by the patient and their physiotherapist. No interviews were held during the rehabilitation sessions. Twelve of the fourteen interviews took place at the Physiotherapy Clinic, with the remaining two conducted at participants’ homes by request.

### Transcription and analysis

Fourteen semi-structured qualitative interviews were recorded and transcribed verbatim using NVivo 12. Data analysis employed Strauss and Corbin’s (1998) open and axial coding methods. During the open coding phase, all data were transcribed verbatim with meticulous attention. Recordings were reviewed multiple times to ensure thorough understanding, particularly when linguistic challenges arose.

#### During Open Coding

 the data was broken down into meaningful segments and labeled with descriptive codes that capture the essence of each piece. This helped identifying key themes and patterns. During Axial coding related codes were categorized into broader themes.

The transcribed data was read through several times to get an overview of the dataset. Coding was guided by Saldan’s coding manual with the assistance of NVivo 12 [18, 19]. Several rounds of coding were conducted by the first author and in collaboration with the third author and a research assistant. Peer coding ensures the quality of the analysis and limits the risk of bias [20]. Several discussions were held to discuss the coding tree with the co-authors.

## Results

A total of 14 participants were included in the study, comprising 12 males and 2 females. Of these, 4 were employed, and 10 were retired. The age at the time of stroke ranged from 46 to 87 years (mean age = 62.43 years). At the time of the interviews, participants were aged between 50 and 88 years (mean age = 65.43 years). The duration since the stroke onset varied from 0.2 to 14 years, with a mean duration of 3.55 years (Tables 1, 2 and 3).

**Table 1:** Inclusion and exclusion criteria

Inclusion criteria:	Exclusion Criteria:
• Understand English	• Unable to understand English
• Ghanaian citizen and/or been born in Ghana.	• Not affected in their mobility
• Physical disability that they define as a physical hindrance related to mobility.	
• Patient at the specific clinic.	

**Table 2:** Study population

Category:	Range:	Mean:
Age at Stroke	46–87 years	62.43 years
Age at interview	50–88 years	65.43 years
Time since Stroke	0.2–14 years	3.55 years

**Table 3:** Study population gender and occupation

Gender	Male 12	Female 2
Occupation	Working 4	Retired 10

Four themes were created from the data analysis: [1] Mobility limitations; [2] Psychosocial burden [3], Employment limitations; and [4] Financial burden.

### Mobility limitations

Limited mobility was mentioned as a major problem for stroke survivors, which affects their quality of life. Generally, participants described Accra as inaccessible for those with disabilities: *“…. Maybe they [the city] should have more for the disabled. People are disabled*,* … but Accra is not disability friendly*,* you know.” [Man *,*40–60 years].* Participants described that they no longer move around the city independently and when doing so rely on assistance. Participants felt incapable of common activities including shopping and visiting a bank (an important task in a context without easy access to internet banking). A common regret was participants*’* exclusion from attending religious services. In the words of one participant: “*I want to go to church*,* unfortunately they haven´t installed the elevator yet. And eeh they have done the walkway*,* but it is steep.” [Woman *,*40–60 years].*

Further, fear was a factor hindering people from moving around and going about their daily activities. For example, participants mentioned the constant fear of getting hit by cars and obstructed by traffic in other ways. *“Well*,* you can’t do a lot of that[walking] because Accra is not safe”* [Man 40–60 years]. Some of the participants lived in gated communities, and these were considered to be safer to walk and move within. These communities had less traffic and well-maintained pavements for walking. However, gated communities were accessible only to a few participants who could afford to live in those areas.

### Psychosocial burden

A second theme that emerged from the analysis was that stroke survivors feel both socially and spiritually isolated, often due to stigma. Participants felt that in the eyes of the community and government, a disabled person is considered worthless. *“Now I am handicapped*,* I have to lie down and get care. I’m handicapped. I cannot do anything”* [Man, 60–80 years]. Stigma was also felt from friends and family. Participants felt that these individuals were tired of the needs and limitations of stroke survivors. *“So*,* when you see the people around you*,* it seems like they are tired*,* they are fed up with you*,* it really*,* really brings you down*,* and it is not easy [Woman 40–60]”*. Social isolation was not only due to post-stroke symptoms, but also due to restricted movement. People had stopped calling, visiting, and inviting them for social activities. Restricted mobility means spending much time alone, and the stigma and expectations surrounding the disease would often result in participants not feeling productive. This affected their mental health negatively.

The inability to perform other activities like washing clothes, cooking food, and cleaning led to isolation, which manifested itself as being constantly treated like a patient by family and friends. *“I would like to do it*,* (cook) but she will not let me. Hmm. She won’t let me do it” [Woman*,* 40–60 years*]. Situations occurred in which people close to the participant undertook tasks, considering it to be an act of kindness- the outcome of this was that the participants felt incapable of living independent lives. Even if they wanted to do the activity, they were not allowed to by family members. Several of the participants expressed that to feel valuable, they needed to work and contribute to the family and society. *“I would say because I used to wash my things. That is my hobby*,* washing. But now I can’t do that” [Man*,* 60–80 years]*. Both the inability to contribute to the household and do things that were considered fun had negative effects on the participants’ overall well-being and mental health. Perceived Incapability showed to easily develop and increase the risk of going into depressive symptoms and a feeling of total lack of hope for the future. The hopelessness of not being able to develop and evolve in life was seen from the participants. “*One thing that every stroke patient have to do is to make uphis or her mind that I´m going to live*,* I’m going to live other than that no amount of physio*,* no amount of drugs*,* you will go!” [Woman*,* 60-80years]*.

A further concern for many was spiritual isolation as a result of limited mobility and social isolation. Churches were seen as important social institutions to attend to meet people and advance socially in Ghana. The limited possibility to attend and be an active part of that social community was worrying for the stroke patients. Participants felt that they could not make spontaneous decisions on how to live their lives after their stroke. This led to making choices not to attend different activities such as church services, spending time with friends and family and other social gatherings. This lack of freedom leads to a negative expressed view of life.

Many churches being handicap inaccessible is a restrictive factor for attending. *“It has really affected me psychologically because I wish I could go to church” [Woman 40–60 years]*. For many participants the inability to attend church anymore negatively impacted their spiritual life, making them unable to practice their religious beliefs.

### Employment limitations

Reintegration into the workplace posed challenges for participants. This was especially burdensome for those who wished to work but at reduced hours, which was not possible. Several of the participants had a feeling of not being able to meet the demands that were put on them. **“***I cannot sustain it (the work hours). Because they go in the morning at 7.30*,* I’m at work*,* and we will be the last people to close at 5 or 6*,* and if the boss don’t leave*,* I can’t leave.” [Man 40–60 years]*.

Additionally, there were experiences of being forced into early retirement against the participants’ will. There was a sense of frustration about the inability to take sick-leave and the long-term ramifications of not being able to work at all. *“I wasn’t happy; I wasn’t happy at all because I wanted to go to work. But they are not waiting for me” [Man 40–60 years]*. The economic consequences of this could be severe since the employer, in some cases, did not take financial responsibility for the retired individuals’ healthcare costs. Furthermore, the pension money was commonly lower than what the participants’ salaries had been. Without gainful employment the economic burden was even greater.

### Financial burden of a stroke

The economic burden following a stroke was a challenge for the survivors and their families. There were huge costs to pay fees for healthcare, including acute care, expensive doctors*´* appointments and physiotherapy. Other costs include paying for hygiene products, and assistive devices like walking sticks, wheelchairs, and handicap adjusted beds. The need for assistance due to mobility barriers led to the cost of hiring nurses and private drivers.

*It is very*,* very difficult. This condition has really drained us. The money which has to go in*,* into paying the physiotherapist*,* paying medication*,* paying to go to health units. It is not easy. Not easy at all. [Woman 40–60 years]*

The lack of economic means was a major burden on families. To take care of the stroke survivors, some spouses stopped working, which ultimately resulted in a reduced family income. There was a general feeling that everything in society was expensive, and you were on your own financially and could not rely on the government for financial support.

Some participants could afford the care but believed that many did not have that privilege: *“You know*,* I have the means. It is not a problem for me*,* monetarily. But a lot of people I know they don’t have the means for a stroke” [Man 40–60 years].*

Health insurance was considered to be an insufficient payment method in Ghana because of the lack of coverage of the insurance. *“No. Because. I haven’t bought it; I don’t think it covers one-tenth of the kind of bills I got” [Man 60–80 years].* Participants believed that it was a hassle to gain insurance because there was a lot of bureaucratic work to manage. Health insurance could also be obtained through the workplace or company, but this was seen as complicated and insufficient since several healthcare fees still needed to be paid out of pocket even with insurance.

## Discussion

This research aimed to understand stroke survivors’ perspectives of living with disabilities in the context of urban Accra, Ghana. The study describes how the specific environment restricts mobility and independence. It also describes stigma, social, and spiritual isolation. The importance of practicing religion echoes previous research on religion and community in Ghana [21]. Participants struggled with reintegrating back into society and working life, thus placing a huge financial burden on the family. The non-medical direct costs, such as adjustments in housing and vehicles, and the indirect costs like the hiring of domestic assistance were significant expenditures described in this study. Health insurance was not seen as an alternative for financial support, and few of the participants were engaged in a healthcare plan. Recent studies have shown that despite the vision that the Ghanaian population would be covered by a health insurance plan, about 40-45% of all direct health expenditures in Ghana were paid from out-of-pocket [8, 22]. Several participants in this study expressed gratitude over being able to pay for their healthcare costs. However, without their savings and financial support from friends and family members, they would have encountered significant problems and may even not be alive anymore. These findings reflect on earlier findings that implies that without family support, it would be challenging for disabled people in Ghana to live valuable lives and reach their full development potential [23, 24]. This finding highlights an urgent need to improve the current understanding of the cost of disability to help accurately devise interventions and policy suggestions to support people living with a disability.

A further finding suggests that self-image and stigma following stroke affect participant*’*s well-being and quality of life. These findings are consistent with earlier studies from Ghana that indicate that a disabled person is seen as “deformed and subhuman” compared to others [25–27], which furthermore leads to a denial of rights in healthcare and public life [25–27]. The analysis presented here further showed that family support was extremely important. According to previous research, the amount of family support and possible avoidance by family members could predict the quality of life among the disabled in Ghana [24, 28]. Stigma and isolation were considered major barriers to reintegration into society. This study adds spiritual isolation to some existing research and highlights that not accessing places of worship can have a serious negative effect on people*’*s well-being [26, 29]. This finding highlights the urgent need to devise policies and interventions to protect disabled people from such stigma and support people towards independence and to accessing public places.

Mental health and potential depression were highlighted in this study as problems experienced by stroke survivors. This echoes research conducted in Ghana indicating a 36.5% incidence of depression symptoms post-stroke compared to 6.7% depression rates in the general population over the age of 50 years [13]. Kutlubaev et al. further indicate that physical disability after stroke is strongly linked to depressive disorders, and it is therefore advised that all patients with a prominent functional disability should be screened for depression [30]. Earlier research implies that the risk for mortality increases due to depression after the stroke both from natural causes, from suicide [31, 32]. Economic means and a strong family network were highlighted as keen motivators to survive post-stroke and are arguably protective factors against poor mental health. This further highlights the importance of not only screening for depression post-stroke but also devising interventions to allow for successful reintegration into society.

Few of the participants in this study returned to work after the stroke. Several participants did not return because family members and employers did not want them to return to a working life. This finding is in line with earlier literature pointing out that there is a cultural view that disabled Ghanaians should be at home, protected from the world [25, 33]. Earlier studies conducted in a west-African setting indicate that reintegration back to work was very low, and as few as 10% of the participants returned to work after the stroke [34, 35]. These numbers could be compared with studies from high-income countries indicating that between 35% and 74.7% returned to work after stroke [36, 37]. The low rate of returning to work can be very negative since stroke survivors who engage in work post-stroke had less impairment than those who did not [35]. Previous research indicates that factors like socioeconomic status and psychological wellbeing can determine the possibility for stroke survivors to return to work [38]. Further research is needed to investigate the possibility of employment post-stroke in Ghana, and if it is more challenging to reintegrate to work for survivors in other parts of the society and different socioeconomic groups.

Social isolation is a direct result of a stroke survivor’s inability to move around the city freely due to the infrastructure not being suitable for people with disabilities. According to former research, 40% of disabled people claim that the physical environment in Ghana limits their ability to travel [39]. Factors such as lack of structures having wheelchair accessibility, absence of canopy walkways, inaccessibility to public toilets, transport, and buildings were highlighted as critical limiting factors [39, 40]. To go on vacations or shorter trips for pleasure are no longer an option after the stroke, shorter errands in the city were usually kept to a minimum. This study supports the above research and highlights poor urban planning and limited accessibility for people living with disabilities. Grischow et al. further highlights that new handicap policies are required and that it is recommended to build more handicap accessible buildings in Ghana [26]. The existing policies are not seen to be effectively implemented, but helpfulness among different people was seen to partly compensate for this. Significant changes in infrastructure in urbanised cities are recommended to promote a more disability-accessible area for the survivors.

## Conclusion

This study underscores the multifaceted challenges faced by stroke survivors living with disabilities in urban Accra. Participants reported significant issues related to mobility, social and spiritual isolation, employment difficulties, and the substantial financial burden associated with post-stroke care. These findings reveal the urgent need for targeted interventions and policy improvements to support rehabilitation, social integration, and economic stability for stroke survivors. A better understanding of these challenges can guide strategies to enhance accessibility, reduce stigma, and promote independence, ultimately contributing to improved well-being and quality of life for those affected.

## Electronic supplementary material

Below is the link to the electronic supplementary material.

Supplementary Material 1

## Data Availability

All request should be directed to the corresponding author.
