# Supplementary material for: Stroke survivors’ perceptions of living with disability in urban Ghana- a qualitative study
Source: BMC Public Health. 2024 Dec 18;24:3504. doi: 10.1186/s12889-024-21030-6 (PMC11657779; doi:10.1186/s12889-024-21030-6)
Supplement: Supplementary file 1 — Supplementary Material 1 [file 12889_2024_21030_MOESM1_ESM.docx]

SEMI-STRUCTURED INTERVIEW GUIDE

INTRODUCTION

Welcome to this interview!

How are you doing today?

Thank you so much for having me and letting me ask this question!

Objectives:

- To explore the perception of how the study participants, experience the physical environment in their local community.
- To explore the perceptions of the possibility for mobility by foot or other transportation in the society for the study participants.
- To explore the disabled stroke survivor’s mobility in performing work or Social activities.
- Like I wrote to you earlier my name is Petter Persson and I'm a physiotherapist and Global health student from Uppsala University in Sweden conducting this interview for my master essay around having **a post-stroke disability looking into mobility in Accra.**
- I will look into if there are any changed connected to mobility after your Stroke?
- I will ask you a few open questions on this subject. Feel free to talk freely. Like I wrote in the information letter I sent you I'm very curious to hear about your perspective on your daily life and your experience connected to mobility.

**Mobility definition:** The ability to move or be moved freely and easily.

- I will give you full confidentiality and everything that you say will stay between us. Your personal information will not be spread.
- You are not obligated to answer any questions if you don't wish to and you can stop the interview at any time.
- The interview approximately Could take about 30 minutes would that be all right for you? Depending on hove much we go into dept of the questions it could also be longer or shorter!
- There are no wrong answers in this interview!
- Is it ok for me to record this interview? I will only use it as a transcript and after that, it will be erased. You have already been informed about this earlier in the written material give to you. Of course, all your answers will be handled anonymously and only heard by me.
- I will also take some small notes during the interview. Feel free to tell me if it is not ok to record then I will write it down instead! Do you have any questions before we start?

**Broad questions**

1. Could you tell me when you first had your stroke?
2. At what age did you have your stroke?
3. Could you briefly describe the day you had your Stroke?
4. Could you describe what form of disability you had past your Stroke?
5. Do you know what type of Stroke you have suffered from?

*1 Section: physical environment:*

1. Has your Mobility and activity performance level changed in any way after you had your Stroke?
   1. And if, in what way?
2. Could you briefly tell me about the first week after the stroke?
   1. How did your recovery proceed?
3. Could you explain how a normal day in your life looks like now?
4. Do you experience any physical limitations in living in Accra after being affected by your stroke?
5. Do you have any experience around difficulties in moving around in your local community post stroke?

2 Section mobility by foot or other transportation:

1. Do you perceive any Physical limitations when walking outdoors In Accra?
   1. What are they due to?
2. Do you experience any physical weakness after your stroke?
3. Could you describe your choice of transportation towards and from work/activities?
   1. Have your choice of transportation changed from before your stroke? (General)
   2. Do you currently drive post stroke?
4. How do you experience commuting with public transportation in Accra?
   1. Has that changed after your stroke?
5. Do you have any fear of falling connected to this?
6. How do you experience commuting to health facilities?
   1. Have that changed after the stroke?
7. Do you currently use any assistive devices to aid with mobility?
   1. How has your experience been with the use of assistive devices?
8. Has your ability to work changed post stroke?
   1. If yes, In what way?

**3 Section work or social activities**

1. How do you experience doing physical household work?
   1. Do you have any help in household work from second part?
   2. How is that help provided for you?
2. Do you currently engage in physical activity or training sessions post stroke?
3. Do you currently engage in any social activities?
   1. If your social life has changed, how have your social life changed after your Stroke?
4. The financial aspect of having a stroke in Ghana, how do you deal with that?

**Summaries:**

- I will try to summaries what you have said during this interview:
- If I understand you correct you say that…………
- Do I understand you correctly?

Thank you very much for this interview!

- I will conduct all these interviews and collect them into a master thesis. Do you accept that I contact you and give you the final version? It is all right also not to.
- If its ok. You could write your number and or email address on this paper?
- The reason for why I do this research is because I want to get a result that I can present to make the situation better for people going through a stroke and live with post Stroke in Ghana. Like I told you earlier, full confidentiality will be obtained from this study.

If you have any further questions, or if you want to withdraw from the study please don't hesitate to call me or send me an email my contact information is at the beginning of the letter. I also would like to ask you if I could contact you if I have any further questions?

## 
